# Anatomical and Functional Changes After Surgical Treatment of Carpal Tunnel Syndrome: A Review

**DOI:** 10.7759/cureus.99904

**Published:** 2025-12-23

**Authors:** Evangelos E Tzanis, John Vlamis, Sophia A Syngouna, Evangelos Sakellariou, Angeliki Kyriakou, Loukia Fragkopoulou

**Affiliations:** 1 3rd Orthopaedic Department, KAT Hospital, National and Kapodistrian University of Athens School of Medicine, Athens, GRC; 2 Department of Hand-Upper Limb and Microsurgery, KAT Hospital, Kifisia, GRC; 3 Department of Radiology, KAT Hospital, Kifisia, GRC

**Keywords:** anatomical changes, carpal tunnel syndrome, conservative surgical, diagnosis, epidemiology, pathophysiology, postoperative recovery, risk factors, symptoms, treatment

## Abstract

Carpal tunnel syndrome (CTS) is a common condition resulting from the compression of the median nerve within the carpal tunnel (CT), leading to sensory and motor deficits in the hand. This review synthesizes current literature on the anatomical and functional changes following surgical intervention for CTS. Surgical treatment, primarily through carpal tunnel release (CTR), has been shown to alleviate symptoms for many patients; however, variability exists regarding the extent of anatomical recovery and functional improvement. Analyzing key studies, we observe that while most patients experience significant relief from pain and sensory symptoms, some may continue to report functional limitations. The prevailing conclusion is that while surgical intervention addresses compression, the extent of anatomical recovery, such as nerve conduction velocity and morphological changes in hand structure, remains underexplored. The evidence indicates that although surgical treatment generally yields positive outcomes, it often falls within the framework of statistical analysis that relies on reported outcomes without rigorous control measures. As such, further controlled studies are necessary to better elucidate the relationship between anatomical alterations and functional recovery post-surgery. The review emphasizes the need for a comprehensive approach in future research that not only focuses on statistical outcomes but also incorporates qualitative assessments of patient functionality and anatomical restoration. Ultimately, a deeper understanding of these domains will enhance the predictive models for recovery and guide clinical practices for individuals suffering from CTS. This analysis seeks to bridge existing gaps and inspire future studies aimed at optimizing therapeutic strategies for CTS.

## Introduction and background

Patients diagnosed with carpal tunnel syndrome (CTS) have been documented in the surgical literature since the mid-1800s. The development of our understanding of CTS is fascinating and significant. From an early age, there was confusion about pathophysiology, resulting in various etiological theories. The clinical findings merged later, resulting in a more precise clinical picture [1]. It is a puzzling and disabling condition commonly presented to rheumatologists and orthopaedic hand clinicians. It is a compressive neuropathy, which is defined as a mononeuropathy or radiculopathy caused by mechanical distortion produced by a compressive force [2]. It now represents a significant problem with a high impact on the quality of life of many patients, with significant socioeconomic consequences. CTS is a condition of nerve entrapment of the upper extremity and is specifically due to the pressure of the median nerve passing through the wrist [3]. Trapping neuropathies and peripheral nerve disorders are defined by the damage and pressure of the nerve passing through a narrow space and occupy the highest position in the list of mononeuropathies [4]. Damage to the median nerve in the carpal tunnel (CT) results in mechanical compression and local ischemia [5].

## Review

Incidence/prevalence

CTS is the most prevalent condition of its kind, a form of nerve entrapment in the upper limb, with a prevalence of approximately 6% [3]. It affects roughly one in 1,000 individuals in the general population [4], 3.8% diagnosed clinically, and 4.9% when diagnosed neurophysiologically [6]. The prevalence of women in many studies is higher than that of men (men-women ratio 1.0:1.4). For women aged 45 to 54 years, the highest symptom of CTS is found, with over 50% of women during pregnancy also showing symptoms of CTS [7]. 

Etiology/risk factors

In most cases, the causes of CTS are not easily identifiable (idiopathic). Secondary causes of CTS include CT lesions such as tumors, hypertrophic joint tissues, fracture calluses, and osteophytes; metabolic and physiological conditions such as pregnancy, hypothyroidism, and rheumatoid arthritis, infections associated with infection, congenital disorders, repetitive activities that require wrist extension or flexion, obesity, fast diet, sleep disorders, and recent menopause [7].

Symptoms and diagnosis

Patients with CTS usually report numbness and altered sensation in the distribution of the median nerve. In addition, some individuals may experience discomfort. These symptoms are often more pronounced at night. In fact, 77% of patients with confirmed CTS, as shown by electromyogram (EMG) testing, experience nighttime numbness or tingling [8-9]. Those who seek treatment later in the disease process may show significant weakness, atrophy of the thenar muscle, and constant paresthesia [10]. CTS can present with subjective signs such as paraesthesia, changes in sensation, and weakness, as well as objective signs including alterations in sensitivity and motor function. Both the Tinel and Phalen tests were positive, and there is noticeable atrophy of the thenar muscles [11]. The diagnosis of CTS relies on classic symptoms such as pain, numbness, tingling, and discomfort in the area served by the median nerve, along with abnormal nerve function. Diagnosing patients with CTS requires the supervising physician's prescription history related to the characteristic signs of the CTS patient. The frequency of these symptoms should be reported, including whether they occur overnight or throughout the day, in particular postures, or due to repetitive movements that trigger the symptoms. Evaluation of the patient's hand is a fundamental approach to diagnosing CTS. The initial medical tests for CTS are the Tinel and Phalen tests [12]. Therefore, patients may be asked to complete a self-diagnosis questionnaire to identify the parts of their hand experiencing the symptoms and classify them as numbness, pain, tingling, or hypoesthesia [13].

Surgery treatment

In the United States, approximately 40% of patients with CTS are treated surgically [12], and in the United Kingdom, 31% of people with CΤS undergo surgery [14]. Long-term studies generally report positive results for CT release (CTR), with a clinical success rate between 75% and 90% [15] CΤS surgery (transverse CT surgery) is usually indicated for people with persistent symptoms who have not previously responded to conservative intervention and have more acute symptoms, such as the frequency of numbness or weakness, or the presence of weakness on the electromyogram, a severe pathological condition [16]. Trials indicate this is a good response from people undergoing surgical treatment compared to splinting [17]. The surgery involves creating an opening in the transverse carpal ligament (TCL) to expand the CT. This procedure decompresses the median nerve. Open surgery and endoscopy are the two approaches commonly used to release the TCL. In open CΤS surgery, the TCL is opened using the palmar surgical incision. With minor open surgery, the technique is done more often with less surgical trauma and shorter recovery time [18]. One goal of CTS surgery is to get the patient back to work or full-time activities as soon as possible. Evaluated the effectiveness of two incisions, one small incision less than 2.5 mm and one greater than 2.5 mm, combined with an active postoperative hand treatment program. Compared to other techniques with or without active physiotherapy, the combined program found that both the short period of return to work and the postoperative complications were fewer [19].

Practical anatomy: CT, features of the transverse ligament, and median nerve

The CT is located at the hand's base (palm) and is partially enclosed by eight carpal bones. It features a hard, fibrous "roof" known as the TCL. The CL serves as a passageway for eight digital flexor tendons, with two tendons for each of the four fingers, as well as a tendon for the long flexor of the thumb (the flexor pollicis longus) and the median nerve. The middle nerve gives sensation by branching into the three fingers (thumb, forefinger, middle) and the middle half [20]. The wrist bones are arranged to form a concave groove in their anterior (palmar) surface, which turns into a tube by the adhesion of the transverse ligament to each of its sides. The wrist joint is a diarthrodial joint composed of eight distinct carpal bones. These bones are situated between the forearm, which includes the radius and ulna, and the five metacarpal bones of the hand. The wrist consists of two rows of carpal bones. The proximal carpal row (PCR) includes, from the radial (thumb) side to the ulnar (pinky) side, the scaphoid, lunate, triquetrum, and pisiform. The distal carpal row (DCR) contains, from radial to ulnar, the trapezium, trapezoid, capitate, and hamate [21]. The average width of the CL at the end of its circumference is 25mm; in its narrow area at the hook level, it is 20 mm, and at the peripheral limit, it is 26mm [22]. Measurements of the transverse ligament length and width correspond to the dimensions of the CL. The maximum depth of the CL is approximately 12 mm, located in the middle of the cross-section, at the ulnar bone level. Each nerve needs to adapt to various positions with passive movement relative to surrounding tissues. The median nerve moves 9.6 mm when the arm is flexed and shifts slightly less during extension [23].

Functional changes

Understanding and treating wrist pathologies requires knowledge of carpal biomechanics, including the role of soft tissues such as ligaments [21]. The TCL’s importance as a pulley in the flexor system, the significance of the transverse fibers of the palmar aponeurosis, and their function in this system have been documented. The flexor tendon excursion available for finger flexion is reduced after open CL release, presumably because of a bowstringing effect on the tendons [24]. Open release divides several structures that endoscopic CL release leaves intact, including the skin and subcutaneous tissues, the longitudinal proximal fibers of the palmar aponeurosis, and the hypothenar and thenar muscle origins [25]. The scientific literature regarding the functional changes, specifically strength and dexterity, following CTR has been limited and contradictory. The patient population studied was diverse, exhibiting a wide range of strength levels; however, they showed substantial declines in the manual dexterity of the affected hand before undergoing CTR. Immediately after the procedure, grip strength decreased by 25%. However, by three months post-surgery, 65% of patients had regained grip strength to pre-operative levels. In addition, grip strength restored to pre-operative levels in 93% of patients when tested on different occasions following the surgery. Although strength and dexterity were assessed using unspecified measures at three months postoperatively, the findings indicated that dexterity had improved, whereas grip strength had not returned to preoperative levels. More recently, a study evaluated the effects of three different surgical techniques on postoperative grip and pinch strength [26-28]. The findings indicated that grip strength scores for all surgical interventions returned to pre-operative levels by 12 weeks post-surgery, although no pre-operative data were provided [29]. However, the strength assessed with the hand dynamometer and finger dynamometer tests shows a marked decline in power grip within the first 2 weeks post-surgery. The affected hand returns to its strength levels six weeks before the surgery [24-30]. In comparing grip and pinch strength before surgery (where the preoperative level is set at 100%) and after the procedure, the following results were observed: Postoperative grip strength was measured at 28% after 3 weeks, 73% after 6 weeks, 99% after 3 months, and 116% after 6 months. For pinch strength, the postoperative values as a percentage of the preoperative mean (which is 100%) were 74% after 3 weeks, 96% after 6 weeks, 108% after 3 months, and 126% after 6 months. Before the operation, grip strength was only 68% of that of the non-involved hand. The postoperative grip strength values were 18% after 3 weeks, 43% after 6 weeks, 57% after 3 months, and 71% after 6 months. However, the authors did not provide standard deviations or any maximum and minimum values, so it is difficult to draw conclusions about the rate of recovery for grip strength [31]. 

Anatomical and morphological changes

Preoperative and Postoperative MRI Analysis

A study utilizing magnetic resonance imaging (MRI) evaluated patients before and six weeks after CTR surgery. The results demonstrated a significant postoperative increase in carpal canal volume. Measurements included carpal arch width, anterior displacement of the canal contents, and total carpal canal volume, assessed using multiplanar reformation (MPR) and three-dimensional MRI reconstructions. Six weeks after surgery, the mean carpal canal volume increased by 24.2 ± 11.6%, representing a statistically significant change (p < 0.001). This widening of the carpal arch persisted at the eight-month follow-up, indicating a sustained anatomical effect of the procedure [32-33].

Measurements of the Carpal Arch Width

The mean carpal arch width before surgery was 24.0 mm. Postoperatively, this increased to 25.5 ± 1.8 mm (p < 0.001), reflecting a favorable structural alteration that may contribute to symptom relief in patients with CTS. In addition, a statistically significant but modest increase of 6.3 ± 4.6% was observed in the distance between the hook of the hamate and the beak of the trapezium six weeks after division of the TCL.

Relationship Between Measurements and Patient Factors

The study further examined the relationship between carpal arch widening and patient-specific factors, including forearm circumference and wrist range of motion. Following surgery, the mean widening of the transverse carpal arch was 10.4%, corresponding to an absolute increase of 2.7 mm. These findings suggest that individual anatomical characteristics may influence postoperative morphological changes and surgical outcomes [32-33].

Long-Term Changes Post-Surgery

According to Peter’s research, additional MRI analyses assessed parameters related to the TCL over a six-month period following open CTR. Significant postoperative changes were observed, including an increase in CT volume from 7.55 ± 1.66 cm³ preoperatively to 8.69 ± 1.66 cm³ postoperatively, representing a 15% increase and supporting the long-term effectiveness of surgical decompression [34].

In patients with CTS, intracarpal tunnel pressures may reach values as high as 100 mmHg during wrist motion. Prolonged exposure to elevated pressure can result in median nerve injury, initially causing sensory demyelination and subsequently progressing to motor involvement and axonal loss.

Microscopic observations in CTS

Histological studies indicate that the primary pathological feature underlying the development and progression of CTS is non-inflammatory fibrosis of the sub-synovial connective tissue (SSCT), particularly surrounding the flexor tendons. This observation underscores the importance of recognizing the structural and histopathological changes contributing to CTS and highlights the relevance of monitoring these alterations following surgical intervention. Collectively, anatomical, biomechanical, and histological findings provide strong evidence for the effectiveness of CTR surgery and its sustained benefits for patients with CTS [35-36-4].

Recommendations for future research

Future research on CTS should focus on longitudinal studies to track functional recovery over time, specifically comparing grip and pinch strength to dexterity. Investigating the differences between open and endoscopic surgical techniques could refine treatment approaches. Standardized postoperative rehabilitation protocols are essential to optimize recovery. In addition, examining biomechanical changes post-surgery will enhance our understanding of the median nerve's environment. Incorporating patient-reported outcomes and exploring early intervention strategies can provide valuable insights. Finally, developing clinical prediction models may identify patients at risk for delayed recovery, tailoring interventions accordingly.

## Conclusions

CTR surgery induces significant anatomical and biomechanical alterations in the CT, resulting in measurable increases in canal volume and median nerve decompression. While short-term postoperative weakness in grip and pinch strength is a common occurrence, likely due to changes in tendon mechanics and soft tissue disruption, most patients experience substantial recovery of strength and improvement in manual dexterity within three to six months. The surgical outcomes are generally favorable, with long-term resolution of symptoms and restored hand function. A thorough understanding of the anatomical changes and functional dynamics following surgery is essential to optimize treatment strategies and rehabilitation protocols for patients with CTS.
